# Field‐Free, Deterministic Giant Spin‐Orbit Torque Switching of 1.3 T Perpendicular Magnetization With Symmetry‐Lifted Topological Surface States

**DOI:** 10.1002/adma.202519678

**Published:** 2025-12-22

**Authors:** He Ren, Yawen Peng, Meixin Cheng, Yu Shi, Reza Asadi, Adam W. Tsen, Guo‐Xing Miao

**Affiliations:** ^1^ Institute For Quantum Computing (IQC) University of Waterloo Waterloo ON Canada; ^2^ Department of Electrical and Computer Engineering University of Waterloo Waterloo ON Canada; ^3^ Department of Chemistry University of Waterloo Waterloo ON Canada; ^4^ Department of Physics and Astronomy University of Waterloo Waterloo ON Canada

**Keywords:** chromium telluride, field‐free SOT, intercalated 2D magnet, self intercalation, spin‐orbit torque, topological insulator

## Abstract

The field‐free control of perpendicular magnetization using spin‐orbit torque (SOT) is a key challenge in spintronics, simplifying design and integration for both memory and logic applications. Unlike conventional heavy metal/ferromagnet heterostructures that already break out‐of‐plane symmetry, thus can readily offer in‐plane switching, realizing deterministic out‐of‐plane switching requires breaking additional in‐plane symmetries. In this study, we demonstrate field‐free switching in a heterostructure composed of a self‐intercalated 2D magnet, Cr_3_Te_4_, and a topological insulator, (Bi_0.75_Sb_0.25_)_2_Te_3_. In this system, the surface states of the topological insulator ensure efficient charge‐to‐spin conversion, but not deterministic on its own. The ordered 2 × 1 self‐intercalation of the perpendicular magnet provides additional symmetry breaking on the interface, rendering a combined unidirectional m (C_s_) symmetry. This synergy allows for extremely strong, field‐free SOT switching of Cr_3_Te_4_ with perpendicular coercivity ≈1.3 T. Unlike exfoliated 2D materials that tend to be single‐crystal, our wafer‐scale deposition naturally nucleates three equivalent types of 2 × 1 sublattices, and therefore the combined SOT switching manifests an apparent three‐fold angular dependence. These findings highlight a promising pathway toward efficient, topological insulator‐based spintronic device and material engineering.

## Graphical abstract

We show a giant, bias‐field free, deterministic, spin‐orbit‐torque switching of perpendicular hard magnets with HC over 1.3 T. By combining the three‐fold 3m symmetry from topological insulator surface states with the rectangular mm2 symmetry from the 2 x 1 intercalation in Cr3Te4, the interface symmetry is significantly reduced into a unidirectional m symmetry and produces the extremely strong, deterministic perpendicular switching.

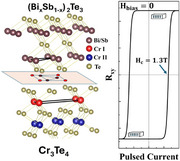

## 1 Introduction

SOT‐driven magnetic switching has drawn a lot of attention in the recent decade due to its high switching speed, low energy consumption, and simple construction [1, 2]. In conventional heavy metal/ferromagnet (HM/FM) based SOT devices, the magnetization of the ferromagnet is switched by the torque from the spin current converted from the charge current via strong spin‐orbit interactions inside the heavy metal. This torque consists of a damping‐like component $\tau_{DL}\propto \hat{m}\times \hat{p}\times \hat{m}$ and a field‐like component $\tau_{FL}\propto \hat{p}\times \hat{m}$, where $\hat{p}$ is the spin polarization direction of the spin current and $\hat{m}$ is the magnetization of the FM [3]. However, this type of torque can only switch FM with in‐plane anisotropy, which has limitations in scalability. In contrast, for a ferromagnet with perpendicular magnetic anisotropy (PMA), the torque τ_*DL*_ cannot deterministically switch the magnetization because the induced in‐plane spin polarization aligns perfectly in the middle between the two PMA states, leading to stochastic final states. To alleviate this in‐plane symmetry, the most common way is to apply an external magnetic field along the charge current direction [1, 4, 5], which adds a torque preferring the magnetization to flip one way rather than the other. The requirement of a bias magnetic field however, adds additional overhead and footprint to the system. In order to realize bias‐field‐free SOT, many efforts have been made, such as engineering tilted anisotropy [6], using antiferromagnets [7, 8], fabricating magnetic trilayers [9, 10], and introducing a lateral structural asymmetry [11]. Another way is to utilize the intrinsic asymmetry in the material itself. To date, field‐free SOT of PMA materials has been demonstrated in several systems with intrinsic low‐symmetry [12, 13, 14, 15]. Related spintronic devices have also been explored [16, 17]. Here we grow a heterostructure of a topological insulator (Bi_x_Sb_1‐x_)_2_Te_3_ (BST), onto a magnetically hard (coercivity ∼1.3 T) PMA material Cr_3_Te_4_, a self‐intercalated transition‐metal dichalcogenide with reduced lattice symmetry. The ordered 2 × 1 intercalation reduces the interface's symmetry to the lowest, unidirectional C_s_ symmetry, while the spin‐locked surface state of BST provides extremely high charge‐spin conversion efficiency. The combined structure manifests an extremely strong field‐free SOT to overcome the large perpendicular coercive force of Cr_3_Te_4_. Further exploration shows that the lower than 100% switching ratio originates from a synergistic contribution of three equivalent domains.

### 1.1 Main

**FIGURE 1:**
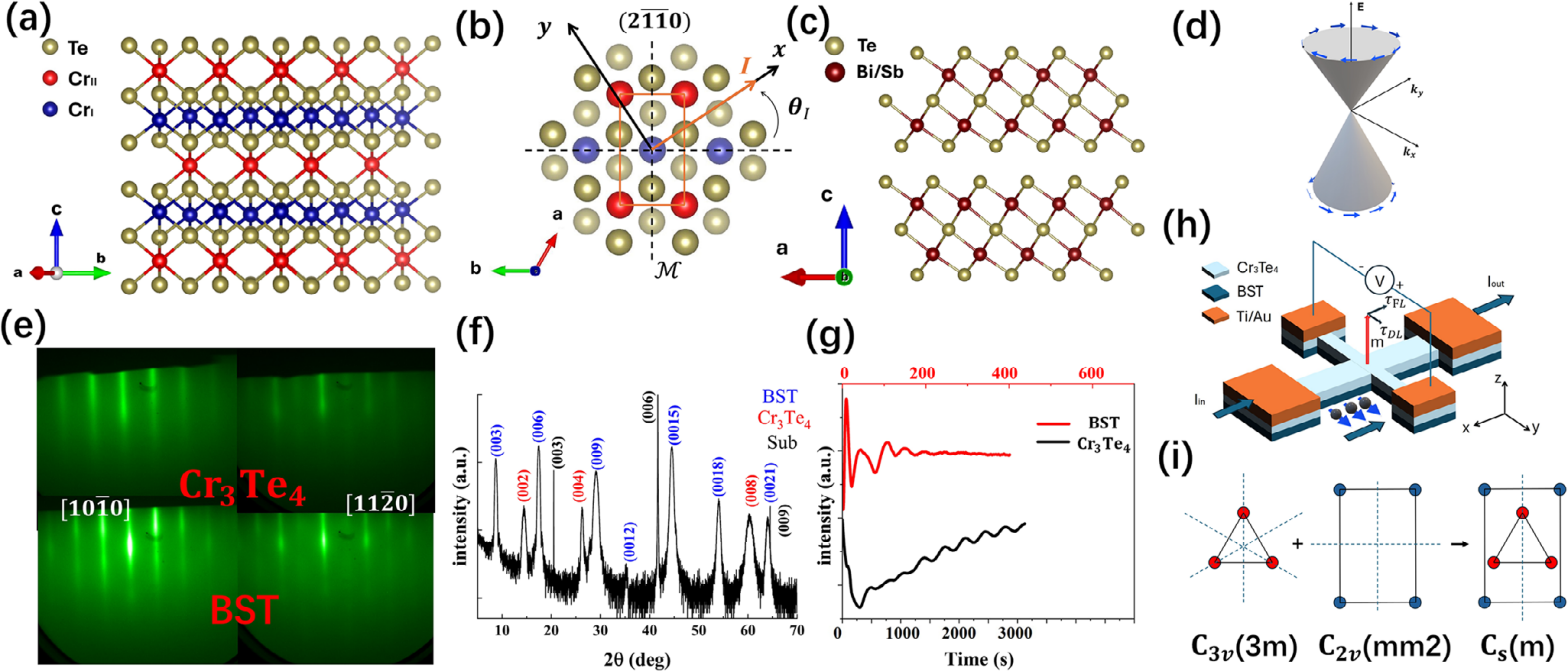
(a) Crystal structure of Cr_3_Te_4_. The intercalated Cr_II_ is labeled by red color. (b) Top view of Cr_3_Te_4_ from the *c*‐axis. Te atoms from the lower layers are shaded for clarity. There is a mirror symmetry with respect to the ($1\bar{2}10$) plane. The symmetry is broken in the other 120° directions due to intercalation. θ_*I*_ is the angle between the direction of pulsed current (labeled as *x*‐axis) and the low symmetry $\left[0\bar{1}10\right]$ direction. The 2 × 1 sublattice is labeled by the orange cell. (c) Crystal structure of (Bi_0.75_Sb_0.25_)_2_Te_3_. Bi atoms can be arbitrarily substituted by Sb atoms. The ratio of the two elements is controlled by the fluxes during the growth. (d) The Dirac cone in k‐space. The direction of spin (blue arrows) is locked for specific $\overset{⇀}{k}$. (e) In situ RHEED pattern after the growth of Cr_3_Te_4_ and the subsequent BST, viewed from $\left[10\bar{1}0\right]$ and $\left[11\bar{2}0\right]$ respectively. (f) XRD result for the heterostructure. Only reflections from *c*‐planes are observed. (g) The RHEED intensity oscillations during the Cr_3_Te_4_ and BST growth. (h) Schematic of the Hall bar device. The current is sent along the $\hat{x}$‐axis and the Hall voltage is measured along the $\hat{\;y}$‐axis. The red arrow is the magnetization, and the black arrows are the field‐like torque and damping‐like torque, respectively. The current flowing on the surface of BST induces an in‐plane spin polarization along the $\hat{y}$ axis. (i) An intuitive illustration of combining space group 3m and 2/m to get a low‐symmetry space group m.

Cr_3_Te_4_ is a self‐intercalated covalent 2D magnet with an out‐of‐plane magnetic anisotropy. The crystal structure of Cr_3_Te_4_ is shown in Figure 1a. Cr atoms (labeled as Cr_II_ in Figure 1a) are intercalated between layers of the parent dichalcogenide compound CrTe_2_, forming a 2 × 1 sublattice. CrTe_2_ by itself is a low‐symmetry system with a space group of $P\bar{3}m1$, where there is a three‐fold rotational symmetry about the z axis, an inversion symmetry, and a mirror symmetry with respect to the ($1\bar{2}10$) plane (and the equivalent {11$\bar{2}$0} planes). Similar systems with reduced symmetry, like WTe_2_ [12, 13, 16] and CuPt/CoPt [14] have been reported to carry field‐free SOT along the low‐symmetry directions. In this work, we use the molecular beam epitaxy (MBE) method to grow Cr_3_Te_4_ few‐layer thin films. The 2 × 1 orderly intercalated atoms in Cr_3_Te_4_ further reduce the lattice symmetry by eliminating the rotational symmetry about the *z*‐axis (as shown in Figure 1b), bringing the new space group into *P*2/*m*. This is analogous to the WTe2 systems where W atoms are also spaced nonuniformly in a 2 × 1 fashion, but the symmetry breaking is now occurring in the SOT receiving layer in our system. Thus, Cr_3_Te_4_ can be a good candidate for harvesting field‐free SOT, even if the originating spin current possesses in‐plane spin polarization.

On the other hand, topological insulators have been reported to be very effective in generating SOT [15, 18, 19, 20, 21] due to their spin‐momentum‐locked surface states (as illustrated in Figure 1d), which provide large charge‐spin conversion efficiency (also known as the spin Hall tangent) $\tan\theta_{SH}=J_{s}/J_{c}\frac{2e}{\hbar }$, where *J_s_* is the spin current density and *J_c_* the charge current density. Some works had shown the ability of topological insulators to switch the magnetization of materials with large perpendicular anisotropy (H_C_ up to 0.7 T) but needing assistance from strong in‐plane bias fields of ≥ 0.3 T [19, 20]. BST is a widely used topological insulator with a hexagonal crystal structure (Figure 1c), which we can epitaxially grow on the Cr_3_Te_4_ films. tan θ_*SH*_ of BST has been reported to be 150–450 at 1.9 K, which is much higher than usual heavy metals [18]. Our results yield a spin Hall tangent of 27.4 at 4.2 K. The Fermi surface can be tuned by the ratio x between Bi and Sb, which can be controlled by their relative fluxes during the growth [22]. When the Fermi surface lies inside the bulk gap, the surface states carry the majority current density, yielding a large tan θ_*SH*_. By combining BST and Cr_3_Te_4_ in one unified heterostructure, reduced symmetry and large charge‐spin conversion efficiency are both present, allowing us to realize an excellent platform for field‐free PMA switching.

## 2 Result and Discussion

Nine monolayers (MLs) of Cr_3_Te_4_ and Eight quintuple layers (QLs) of (Bi_0.75_Sb_0.25_)_2_Te_3_ were grown epitaxially on sapphire (0001) substrates (see Methods). The thickness and quality of the heterostructures were characterized by in situ reflection high‐energy electron diffraction (RHEED) (Figure 1e) and X‐Ray diffraction (XRD) (Figure 1f). The lattice constants were determined to be *c_CT_* =  1.242 nm and *c_BST_* =  3.081 nm from the θ‐2θ XRD scan.

**FIGURE 2:**
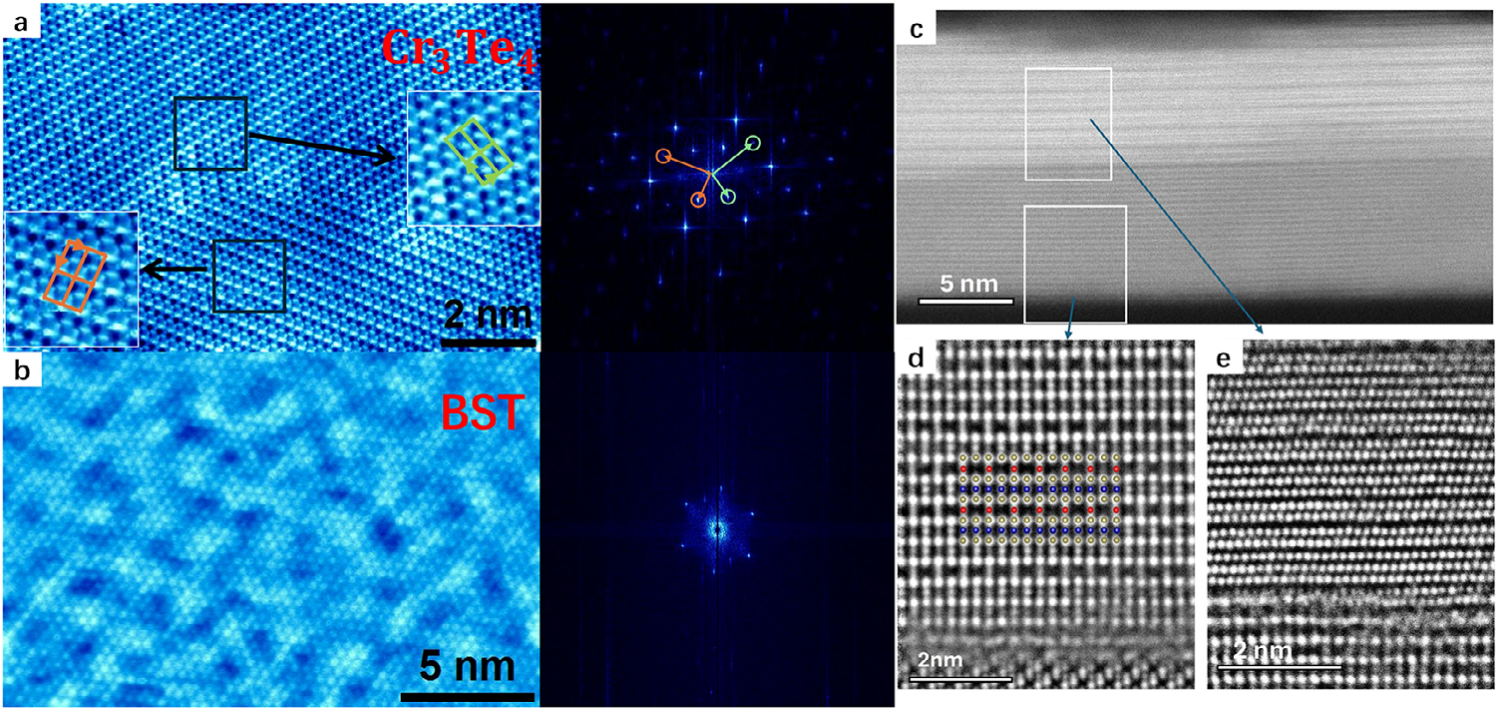
(a) The STM image and corresponding FFT of Cr_3_Te_4_ grown on (0001) sapphire. Two domains that are aligned 120° with respect to each other can be observed clearly in the area. The 2 × 1 sublattices in different orientations are marked by the green and orange boxes in the magnified insets. The corresponding superlattice symmetry points are also marked in the FFT figure and Figure 1b. (b) (Bi_0.75_Sb_0.25_)_2_Te_3_ grown on top of Cr_3_Te_4_. It shows a simple hexagonal lattice. (c) Cross‐sectional HAADF‐STEM image of the heterostructure along [21$\bar{3}$0] zone axis. (d) The enlarged view of the HAADF image at the interface between Cr_3_Te_4_ and the substrate. The corresponding Te, Cr_I,_ and Cr_II_ atoms are marked with the same colors as in Figure 1a. The occupancy is consistent with the atomic structure model. (e) The enlarged view of the HAADF image at the interface between (Bi_0.75_Sb_0.25_)_2_Te_3_ and Cr_3_Te_4._

To study the surface symmetry of Cr_3_Te_4_, scanning tunneling spectroscopy (STM) was performed (Figure 2a). We can clearly observe the 2 × 1 superlattice of the intercalated Cr_II_ atoms. However, the orientation of the 2 × 1 cells cannot be controlled during growth. There are three equivalent 2 × 1 stacking orientations with identical energies and initiated from the very early nucleation stage. The short edges of the 2 × 1 cells are aligned along the {100} planes, 120° apart from one another. As a result, the sublattices occur with equal probabilities along the three directions. On the right of Figure 2a is the fast Fourier transform (FFT) image of the presented area, which shows two 2 × 1domains that are 120° rotated with respect to each other (the 2 × 1 diffractions are marked by the green and red circles). In larger areas, three 2 × 1 domains can coexist (see Figure S3). We also performed STM on the surface of (Bi_0.75_Sb_0.25_)_2_Te_3_ that was grown on Cr_3_Te_4_. Figure 2b shows the surface image, and the corresponding FFT confirms the expected hexagonal structures. The atomic structure was further characterized by high‐angle annular dark‐field aberration‐corrected scanning transmission electron microscopy (HAADF‐STEM). Figure 2c shows a cross‐sectional overview of the heterostructure. We can clearly observe the Te‐Bi(Sb)‐Te‐Bi(Sb)‐Te quintuple layer of BST. Figure 2d,e show enlarged views of Cr_3_Te_4_ and BST structures in the rectangular areas in Figure 2c. The Cr_II_ atoms (marked as red circles) occupy half of the intercalation layer while the Cr_I_ atoms (marked as blue circles) fully occupy the sandwich layer, further confirming the 3:4 stoichiometric ratio.

**FIGURE 3:**
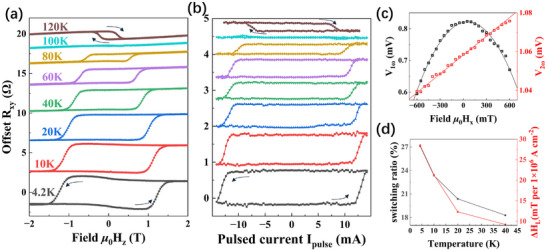
(a) Field switching *R_xy_* hysteresis loops at different temperatures. A hump can be observed near the coercivity at 4.2 K. The sign of AHE is reversed above 100 K. (b) Pulsed current induced *R_xy_* hysteresis loops under zero bias field at different temperatures. It shows similar behavior as field switching. (c) First (black) and second (red) harmonic signals with 5 mA ac current at 4.2 K. The solid lines are the corresponding parabolic and linear fitting, respectively. (d) The trend of the switching ratio and damping‐like effective field Δ*H_DL_* with temperature.

**FIGURE 4:**
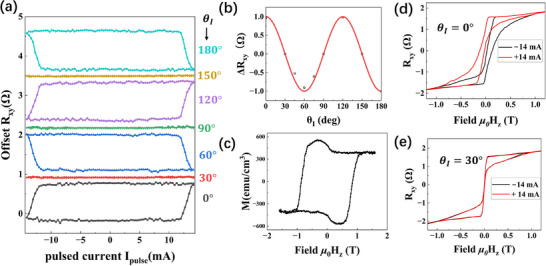
(a) The field‐free SOT measurements with different azimuthal angle θ_*I*_. (b) The relation of Hall amplitude Δ*R_xy_* vs. θ_*I*_. The red line is a fitting with the cosine function. It shows a 120° periodicity owing to the equally populated three 2 × 1 domain types. (c) The magnetization hysteresis curve under a perpendicular magnetic field measured by VSM. (d) and (e) Hysteresis loop shift under 14 mA dc pulses along θ_*I*_ =  0° and θ_*I*_ =  30°, respectively.

The heterostructures were then fabricated into 6 µm × 15 µm Hall bars with different orientations with respect to the $\hat{a}$ ‐axis of the crystal (Figure 1h). The direction of the current is set as the $\hat{x}$ direction, and the angle between the current direction and the $\bar{b}$‐axis is defined as θ_*I*_. The out‐of‐plane anisotropy was first confirmed by field‐dependent Hall measurement (Figure 3a) and vibrating sample magnetometer (VSM) measurement (Figure 4c). The coercivity of the film is determined to be around 1.3 T at 4.2 K. In the Hall measurement, a noticeable hump at 4.2 K can be observed near the coercivity. Another feature is that the hysteresis loop changes from counterclockwise to clockwise as temperature increases past around 100 K, indicating another component starts to dominate the overall AHE response. This phenomenon has been studied in the self‐intercalated Cr_1+x_Te_2_ family and may stem from the sign reversal of Berry curvature caused by the change of strain near the substrate [23], or by Weyl‐like nodes due to the modulated canting angles of the Cr intercalants [24].

Then we applied a sequence of dc pulses with pulse width τ_*pulse*_ =  100 ns through the Hall bar along the $\hat{x}$‐axis and measured the Hall resistance for different temperatures with zero bias field (Figure 3b). The hysteresis loops are observed, confirming the SOT‐driven field‐free magnetization switching. The switching current density is around 1.5 × 10^7^ A cm^−2^ at 4.2 K and decreases gradually to 4.5 × 10^6^ A cm^−2^ at 120 K. A sign reversal of AHE polarity is also observed around 100 K, which agrees with the field‐dependent Hall measurement.

The damping‐like torque can be quantitatively evaluated by taking the second harmonic Hall voltages. We applied a 5 mA ac current at 17.77 Hz along the longitudinal *x* direction of the Hall bar device under an in‐plane magnetic field and obtained the damping‐like effective field Δ*H_DL_* by measuring the first (*V*_ω_) and second (*V*_2ω_) harmonic signals of the Hall voltage [25]:

(1) $$\begin{array}{c} \tau_{X\left(Y\right)}=-2\frac{\partial V_{2\omega }}{\partial H_{X\left(Y\right)}}/\frac{\partial^{2}V_{\omega }}{\partial H_{X\left(Y\right)}^{2}} \end{array}$$

(2) $$\begin{array}{c} \Delta \;H_{DL}=\frac{\tau_{X}+2\xi \tau_{Y}}{1-4\xi^{2}} \end{array}$$

where ξ is the ratio of the planar Hall voltage to the anomalous Hall voltage. The planar Hall voltage correction ξ is usually much smaller than 1 (∼0.04) [26], so Δ*H_DL_* ≈ τ_*X*_. The fittings of *V*_ω_ and *V*_2ω_with quadratic and linear functions are shown in Figure 3c. The damping‐like effective field Δ*H_DL_* of our device is determined to be 28.5 mT per 1 × 10^6^A cm^−2^ at 4.2 K. We can also estimate the spin Hall angle tangent $\tan\theta_{SH}=J_{s}/J_{c}\frac{2e}{h}$ by using the following formula [4, 27]:

(3) $$\begin{array}{c} \Delta \;H_{DL}=\frac{J_{s}\mu_{B}}{\gamma eM_{s}t} \end{array}$$

where μ_*B*_ is the Bohr magnetron, γ is the gyromagnetic ratio of the electron, *M_s_* is the saturated magnetization, and *t* is the thickness. $M_{s}\;∼\;396\;\mathrm{emu}/cm^{3}$ can be obtained from the magnetization curve in Figure 4c. tan θ_*SH*_ in our sample at 4.2 K turns out to be 27.4, which is comparable to the reported values for BST [18]. We also calculated Δ*H_DL_* at other temperatures as shown in Figure 3d. Δ*H_DL_* gradually decreases as the temperature ramps up. Notably, the switching ratio, defined as the ratio between saturation *R_xy_* of bias‐free SOT switching and that of pure field switching, also decreases as temperature increases. This is because the bulk conductance of BST starts to become stronger as the temperature increases due to thermal excitations. The current flow on the surface states is shunted by the bulk flow, which results in the overall lower charge‐spin conversion efficiency and switching ratio of the FM layer.

In order to examine the system's field‐free switching capability, we fixed the temperature at 4.2 K and performed field‐free SOT measurement with currents aligned at different in‐plane angles with respect to the crystal axis $\hat{a}$ (by patterning Halls bars in different orientations on the same film). Figure 4a shows the hysteresis loops with different θ_*I*_. For θ_*I*_ =  120°, the magnetization can be deterministically switched, and the hysteresis loop behaves the same as θ_*I*_ =  0°. For θ_*I*_ =  60° and 180°, the magnetization can also be switched, but in an opposite direction. And for θ_*I*_ =  30°, 90°, and 150°, no clear switching can be observed. By plotting the maximum Hall resistance Δ*R_xy_* against the angles (Figure 4b), we found a three‐fold symmetry.

To fully understand this apparent threefold symmetry under field‐free switching, we analyze the overall structural symmetry in our system. The BST in our device adopts $R\bar{3}m$. The inversion symmetry of point group $\bar{3}m\;$ can be broken at the interface [14] and turns into 3*m*. As a result, a 3*m* torque that allows out‐of‐plane spin component emerges and leads to deterministic switching of the magnetic layers with three‐fold symmetry. Also, a hexagonal spin warping has been found on the surface of many topological insulators [28, 29, 30]. In order to verify whether only the surface state is responsible for the field‐free SOT, we conducted the same SOT measurement on BST (9 QL)/CoFeB (1 nm)/MgO (2 nm) samples, in which CoFeB has PMA but much lower coercivity than Cr_3_Te_4_. However, we did not observe any signs of field‐free SOT switching in both the high‐symmetry and low‐symmetry directions (See Figure S5), while field‐assistant switching was observed with a bias field along the current direction. This rules out the possibility that the BST surface state alone can induce field‐free switching and suggests additional symmetry breaking is in play. Another control sample Pt (2 nm)/Cr_3_Te_4_ (9 ML) was also measured using the same conditions. No SOT switching was observed either with or without bias field before the device breaking down (See Figure S6), indicating a much stronger SOT originating from BST is necessary.

In our system, both the parent compound of Cr_3_Te_4_ and BST adopt a space group of $\bar{3}m$. At the interface, the inversion symmetry will be lifted, resulting in a symmetry of 3*m*. Now adding the intercalated superlattice with a space group of *mm*2, the combined result becomes extremely low in symmetry, only *Pm* (See Figure 1i). Our simulation shows that such low‐symmetry is able to induce an out‐of‐plane torque and hence field‐free SOT (See Figure S1). However, Hall effect from one single‐crystal domain is not expected to have in‐plane rotational symmetry. The explanation is that our films contain three types of randomly distributed domains, 120° apart from one another, with typical domain sizes on the order of 10s nm (Figure 2a). Therefore, over the Hall device area measured, there are millions of such domains equally distributed in 3 directions. On average, the overall behavior of the composite system still manifests an apparent in‐plane three‐fold symmetry. This can also explain the lower than 100% switching ratio in our devices. When a pulse current is applied with an angle θ_*I*_ =  0° with respect to one type of domains, it is actually 120° and −120° to the other two types of domains. While the first domain type is fully switched, the other two are only partially switched due to different orientations, and the overall switching ratio depends on the average from all domains. It is worth pointing out that this combined contribution cannot be solely determined from symmetry and can assume any percentage from 0–100% depending on the physical system of interest. A few examples of different possible z‐magnetization (by varying symmetry coefficients) are shown in Figure S2.

Next, we evaluate the strength of the out‐of‐plane SOT effective field by measuring the magnetoresistance hysteresis loop in the presence of SOT currents [11]. Figure 4d,e show the hysteresis loops under positive and negative 14 mA pulsed current along the low‐symmetry direction θ_*I*_ =  0° and the high‐symmetry direction θ_*I*_ =  30°. The loop of the θ_*I*_ =  0° sample shows a clear shift between the two sweep directions, while the θ_*I*_ =  30° sample shows no difference. The corresponding out‐of‐plane effective field for θ_*I*_ =  0° is estimated to be 80 mT, which is an order of magnitude higher than other reported values [14, 31].

**FIGURE 5:**
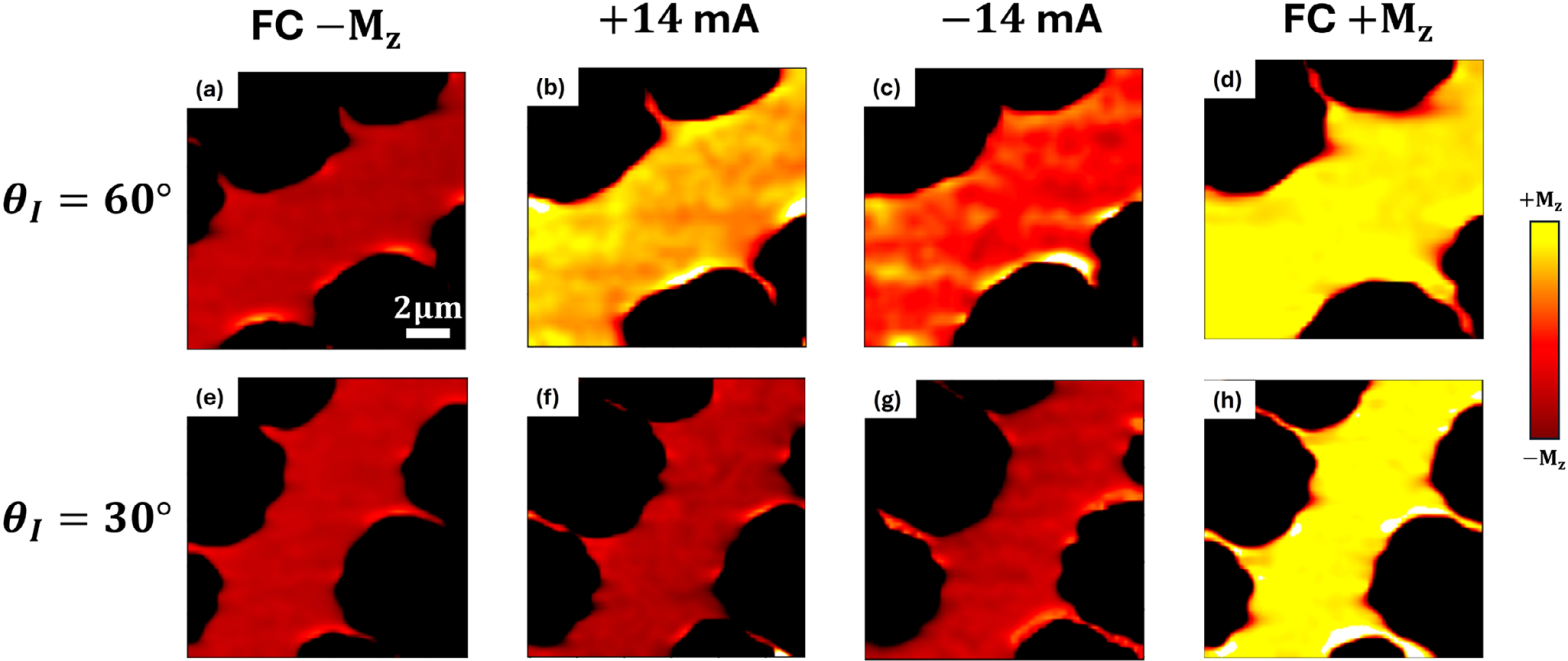
MOKE images of current‐induced field‐free switching. (a,e) Initialized ‐*M* state by negative field cooling in samples with θ_*I*_ =  60° and 30°. (b,f) Sending one 1 µ*s* pulse of 14 mA across the channel. The brighter color corresponds to more magnetization pointing up. (c,g) Sending one 1 µ*s* pulse of −14 mA after the positive pulse. (d,h) Reference +*M* state prepared by positive field cooling.

Magneto‐optic Kerr effect (MOKE) was further measured on the samples with θ_*I*_ =  30° and 60°. The results are shown in Figure 5. The samples were first prepared in a fully magnetized − *M* state by cooling down under a negative out‐of‐plane field (Figure 5a,e). Then a positive +14 mA pulse current with 1 µs was applied in the absence of a magnetic field. As we can see in the second column, only the sample with θ_*I*_ =  60° has a significant change, while the one with θ_*I*_ =  30° remains almost the same. However, the sample is only partially switched as indicated by the intermediate MOKE intensity. As a reference, the fully magnetized + *M* states set by external fields are shown in the last column for each sample. Then, negative −14 mA pulses were sent through the devices. The 60° device was partially switched into − *M* state, and 30° device showed no substantial change, which is consistent with our transport results.

## 3 Conclusion

In conclusion, we demonstrated field‐free, deterministic switching of a PMA magnetic material Cr_3_Te_4_ by giant SOT from topological insulator surface states. The low symmetry interfacial structure enables the switching to happen without needing an external bias magnetic field. The topological surface states with large charge‐spin conversion efficiency, together with the very low interfacial symmetry induced from ordered intercalation, make it possible to switch PMA materials with very large coercivity over 1T. We also highlight the importance of thin film growth and potential multiple domain complications in the SOT applications. These features make the system simple in construction and robust against errors, thus facilitate scalable applications in modern spintronic devices.

## 4 Methods

### 4.1 Sample Growth

The growth of Cr_3_Te_4_/(Bi_0.75_Sb_0.25_)_2_Te_3_ heterostructures was carried out in an MBE system under ultra‐high‐vacuum (UHV) with a base pressure of 10^−10^ Torr. High‐purity Cr (99.99%), Bi (99.999%), and Sb (99.999%) were thermally evaporated from Knudsen effusion cells. Te (99.9999%) was evaporated from a valved cracker cell to avoid large clusters. c‐cut sapphire wafers were used as substrates. Prior to the growth, the substrates were solvent cleaned and in situ annealed at 700°C for 1 h. During the growth of Cr_3_Te_4_, the substrates were kept at 400°C, and Cr:Te flux ratio was adjusted to 1:10. The growth rate was approximately 0.002 nm/s^−1^, which was verified by ex‐situ X‐ray reflectometry. For the subsequent growth of (Bi_0.75_Sb_0.25_)_2_Te_3_, the substrates were cooled to 280°C, and the Bi:Sb:Te flux ratio was adjusted to 3:1:20. The growth rate was approximately 0.01 nm s^−1^. The epitaxial growth of both layers was monitored by in situ RHEED operated at 15 kV. After the growth, the films were annealed at 280°C for 20 min and capped in situ with 8 nm Al_2_O_3_ to protect the heterostructures for ex‐situ fabrication and measurement.

### 4.2 Device Fabrication

The Cr_3_Te_4_/(Bi_0.75_Sb_0.25_)_2_Te_3_ bilayers were patterned, by maskless optical lithography, into a typical Hall bar geometry of 6 µm wide and 15 µm long with different orientations with respect to the $\hat{a}$‐axis defined by the flat of the wafer. Ar ion milling was used for etching. Finally, Ti (15 nm)/Au (50 nm) were deposited as electrodes by e‐beam evaporation.

### 4.3 STM Measurement

The Cr_3_Te_4_ samples were capped in situ by 5 nm Te at room temperature and then transferred to a nitrogen glove box without exposing to air, where the sample was sealed in nitrogen. The samples were then transferred into the Omicron UHV STM, bag opening and sample loading all in the nitrogen‐flushed load‐lock chamber, with no more than a few seconds exposure before pumping down. The samples were first annealed at 350°C to decap the Te protection layer and then cooled down to 4.2 K with liquid helium. The STM images were taken under 1.2 nA and 0.2 V bias.

### 4.4 SOT Measurements

The electrical measurement was carried out in a home‐made Janis 12 T cryostat. A Keithley 6221 current source and a Keithley 2182A nanovoltmeter were used for measuring the AHE hysteresis loop, and square dc pulses of 100 ns were generated by a Keithley 4200A parameter analyzer integrated with ultra‐fast pulse measurement units (PMU). The pulse started from 0 to 40 V and swept a full loop between 40 and −40 V in 1 V steps. The SOT current was measured in the middle of the pulses. The Hall resistance was measured with another smaller excitation current (100 µA) 5 s after each pulse.

### 4.5 MOKE Measurement

MOKE measurements were performed in a Montana C2 cryostat at 22 K. A 520 nm wavelength diode laser was focused onto the samples with a beam spot of ∼1μm using an objective lens of *NA*  =  0.55. The incident light was modulated between left‐ and right‐handed circular polarization by a photo‐elastic modulator (PEM; Hinds Instruments PEM‐200) at 50.1 kHz. The reflected signal was collected by the same lens and detected by a photodiode and two lock‐in amplifiers: one tuned at 100.2 kHz to detect the Kerr signal, and one tuned at the chopper's frequency, 800 Hz, to normalize the Kerr signal. DC pulses of 1 µs were used for inducing the SOT switching.

### 4.6 Second Harmonic Measurement

The first and second harmonic signals were taken by a SR830 lock‐in amplifier. The 5 mA ac bias current was applied by a Keithley 6221 current source.

## Author Contributions

Guo‐Xing Miao conceptualized and supervised the project. He Ren performed the materials deposition, device fabrication, VSM, and transport measurement. Yawen Peng and He Ren performed STM. Meixin Cheng and He Ren performed MOKE. Yu Shi assisted in parameter analyzer programing. R.A. assisted with some transport measurements. He Ren wrote the original draft, He Ren, Yawen Peng, and Guo‐Xing Miao performed the data analysis and manuscript writing, and all authors contributed to manuscript editing.

## Conflicts of Interest

The authors declare no conflicts of interest.

## Supporting information




**Supporting Information File 1**: adma71856‐sup‐0001‐SuppMat.docx

## Data Availability

The data that support the findings of this study are available from the corresponding author upon reasonable request.
