# Supplementary material for: Field‐Free, Deterministic Giant Spin‐Orbit Torque Switching of 1.3 T Perpendicular Magnetization With Symmetry‐Lifted Topological Surface States
Source: Adv Mater. 2025 Dec 22;38(10):e19678. doi: 10.1002/adma.202519678 (PMC12910540; doi:10.1002/adma.202519678)
Supplement: Supplementary file 1 — Supporting Information File 1: adma71856‐sup‐0001‐SuppMat.docx [file ADMA-38-e19678-s001.docx]

**Supplementary Information for**

**Field-Free, Deterministic Giant Spin-Orbit Torque Switching of 1.3 T Perpendicular Magnetization with Symmetry-Lifted Topological Surface States**

He Ren^1,2^, Yawen Peng^1,2^, Meixin Cheng^1,3^, Yu Shi^1,2^, Reza Asadi^1,4^, Adam W. Tsen^1,3,4^, Guo-Xing Miao^1,2,4*^

^1^Institute for Quantum Computing (IQC), ^2^Department of Electrical and Computer Engineering, ^3^Department of Chemistry, ^4^Department of Physics and Astronomy, University of Waterloo, Waterloo, ON N2L 3G1 CANADA

*Email: [guo-xing.miao@uwaterloo.ca](mailto:guo-xing.miao@uwaterloo.ca)

**SI session 1: Numerical simulation of SOT with 3m and m symmetry**

According to Kubo linear response formalism, the current-induced spin polarization $\delta\mathbf{S}$ can be expressed as $\delta\mathbf{S}=\chi\mathbf{E}$, where $\mathbf{E}$ is the electric field and $\chi$ is the response tensor [1]. $\chi$ can be expanded into the first order in Einstein summation notation as [2]:

$$\begin{aligned} \chi_{ij}=\chi_{ij}^{0}+\chi_{ij,k}^{1}\hat{m}_{k}\#\left( AUTONUMLGL \backslash* Arabic \backslash e \right) \end{aligned}$$

where $\hat{m}$ is the unit vector of spin direction. The term $\chi^{0}$ is related to the field-like torque.

For a crystal with $3m1$ point group, there is a three-fold rotational symmetry and a mirror symmetry. Applying these symmetries and considering $\mathbf{E}$ only in the x-y plane, $\chi^{1}$ can be written as:

$$\begin{aligned} \chi^{1}=\chi_{DL}X_{1}+\chi_{3m}X_{2},\#\left( AUTONUMLGL \backslash* Arabic \backslash e \right) \end{aligned}$$

where

$$\begin{aligned} X_{1}=\left( \begin{matrix} \hat{m}_{z} & 0 & 0 \\ 0 & \hat{m}_{z} & 0 \\ {-\hat{m}}_{x} & -\hat{m}_{y} & 0 \end{matrix} \right)\#\left( AUTONUMLGL \backslash* Arabic \backslash e \right) \end{aligned}$$

$$\begin{aligned} X_{2}=\left( \begin{matrix} \hat{m}_{y} & \hat{m}_{x} & 0 \\ \hat{m}_{x} & -\hat{m}_{y} & 0 \\ 0 & 0 & 0 \end{matrix} \right)_{,}\#\left( AUTONUMLGL \backslash* Arabic \backslash e \right) \end{aligned}$$

Then the torque can be expressed as:

$$\begin{aligned} \mathbf{T}=&\boldsymbol{m}\times\delta\mathbf{S} \\ \mathbf{=&}\chi_{\mathrm{DL}}\boldsymbol{m}\times[\left( \mathbf{z}\boldsymbol{\times}\mathbf{E} \right)\times\boldsymbol{m}\boldsymbol{]}+\chi_{3m}\boldsymbol{m}\boldsymbol{\times}\left[ \left( m_{y}E_{x}+m_{x}E_{y} \right)\mathbf{x}\mathbf{+}\left( m_{x}E_{x}\mathbf{-}m_{y}E_{y} \right)\mathbf{y} \right] \\ =&\chi_{DL}\mathbf{T}_{\mathrm{DL}}+\chi_{3m}\mathbf{T}_{3m}\#( AUTONUMLGL \backslash* Arabic \backslash e )\#\#\#\# \end{aligned}$$

The first term corresponds to the effective field for damping-like torque and the second term is unique for $3m$ point group, which we call it 3m torque. $\chi_{DL}$ and $\chi_{3m}$ are the response coefficient for the damping-like and $3m$ torque. The magnetization should follow the equation:

$$\begin{aligned} \frac{d\mathbf{m}}{dt}=\frac{\gamma}{M_{s}}(\chi_{DL}\mathbf{T}_{\mathrm{DL}}+\chi_{3m}\mathbf{T}_{3m})\#\left( AUTONUMLGL \backslash* Arabic \backslash e \right) \end{aligned}$$

When equilibrium is established, the total torque should be zero. We can explicitly write the torque as

$$\begin{aligned} \mathbf{T}\mathbf{=}E\left( \begin{matrix} -\chi_{DL}\left( m_{x}m_{y}\cos\left( \varphi\right)+{(m}_{y}^{2}+m_{z}^{2})\sin\left( \varphi\right) \right)+\chi_{3m}m_{z}\left( -m_{x}\cos\left( \varphi\right)+m_{y}\sin\left( \varphi\right) \right) \\ \chi_{DL}\left( {(m}_{x}^{2}+m_{z}^{2})\cos\left( \varphi\right)+m_{x}m_{y}\sin\left( \varphi\right) \right)+\chi_{3m}m_{z}\left( m_{y}\cos\left( \varphi\right)+m_{x}\sin\left( \varphi\right) \right) \\ \chi_{DL}m_{z}\left( -m_{y}\cos\left( \varphi\right)+m_{x}\sin\left( \varphi\right) \right)+\chi_{3m}\left( \left( m_{x}^{2}-m_{y}^{2} \right)\cos\left( \varphi\right)-2m_{x}m_{y}\sin\left( \varphi\right) \right) \end{matrix} \right)\boldsymbol{\#}\left( AUTONUMLGL \backslash* Arabic \backslash e \right) \end{aligned}$$

where $\varphi$ is the angle between applied electrical field $\mathbf{E}$ and the x axis (low symmetry axis). In order to simplify the case, we assume $\frac{\chi_{3m}}{\chi_{DL}}\ll1$ since $\chi_{3m}$ is only induced by the crystal asymmetry. As a result, $m_{z}$ should be close to zero. Under these approximations, the torque becomes:

$$\begin{aligned} \mathbf{T}=E\left( \begin{matrix} -\chi_{DL}m_{y}\left( m_{x}\cos\left( \varphi\right)+m_{y}\sin\left( \varphi\right) \right) \\ \chi_{DL}m_{x}\left( m_{x}\cos\left( \varphi\right)+m_{y}\sin\left( \varphi\right) \right) \\ \chi_{3m}\left( \left( m_{x}^{2}-m_{y}^{2} \right)\cos\left( \varphi\right)-2m_{x}m_{y}\sin\left( \varphi\right) \right) \end{matrix} \right) \\ \boldsymbol{=}\chi_{DL}\left( \mathbf{m}\boldsymbol{\cdot}\mathbf{E} \right)\left( \mathbf{z}\boldsymbol{\times}\mathbf{m} \right)\boldsymbol{+}\chi_{3m}Em_{0}^{2}\sin\left( 2\varphi_{m}-\varphi\right)\mathbf{z}\boldsymbol{\#}\left( AUTONUMLGL \backslash* Arabic \backslash e \right) \end{aligned}$$

where $m_{0}$ is the in-plane magnetization and $\varphi_{m}$ is the angle of in-plane magnetization with respect to the x axis. To make $\mathbf{T=0}$, $\mathbf{m}$ has to take the form $(-m_{0}sin\varphi,m_{0}cos\varphi,m_{z})$ assuming $\chi_{DL}>0$. Putting $\mathbf{m}$ back to $( REF equ\_7 \backslash h \backslash* MERGEFORMAT 7)$, and we can solve the equation at the equilibrium and get:

$$\begin{aligned} m_{z}\left( \varphi\right)=-\frac{\chi_{3m}}{\chi_{DL}}m_{0}\cos\left( 3\varphi\right).\#\left( AUTONUMLGL \backslash* Arabic \backslash e \right) \end{aligned}$$

The $m_{z}$ at equilibrium has a three-fold symmetry.

In order to validate the result obtained from approximation, we also solve the equation $( REF equ\_6 \backslash h \backslash* MERGEFORMAT 6)$ numerically. As shown in **Figure S1**, the final $m_{z}$ follows exactly $\cos\left( 3\varphi\right)$ while the in-plane component has a periodicity of $2\pi$. The inset of Figure S1(a) shows the evolution of each component of $m$.


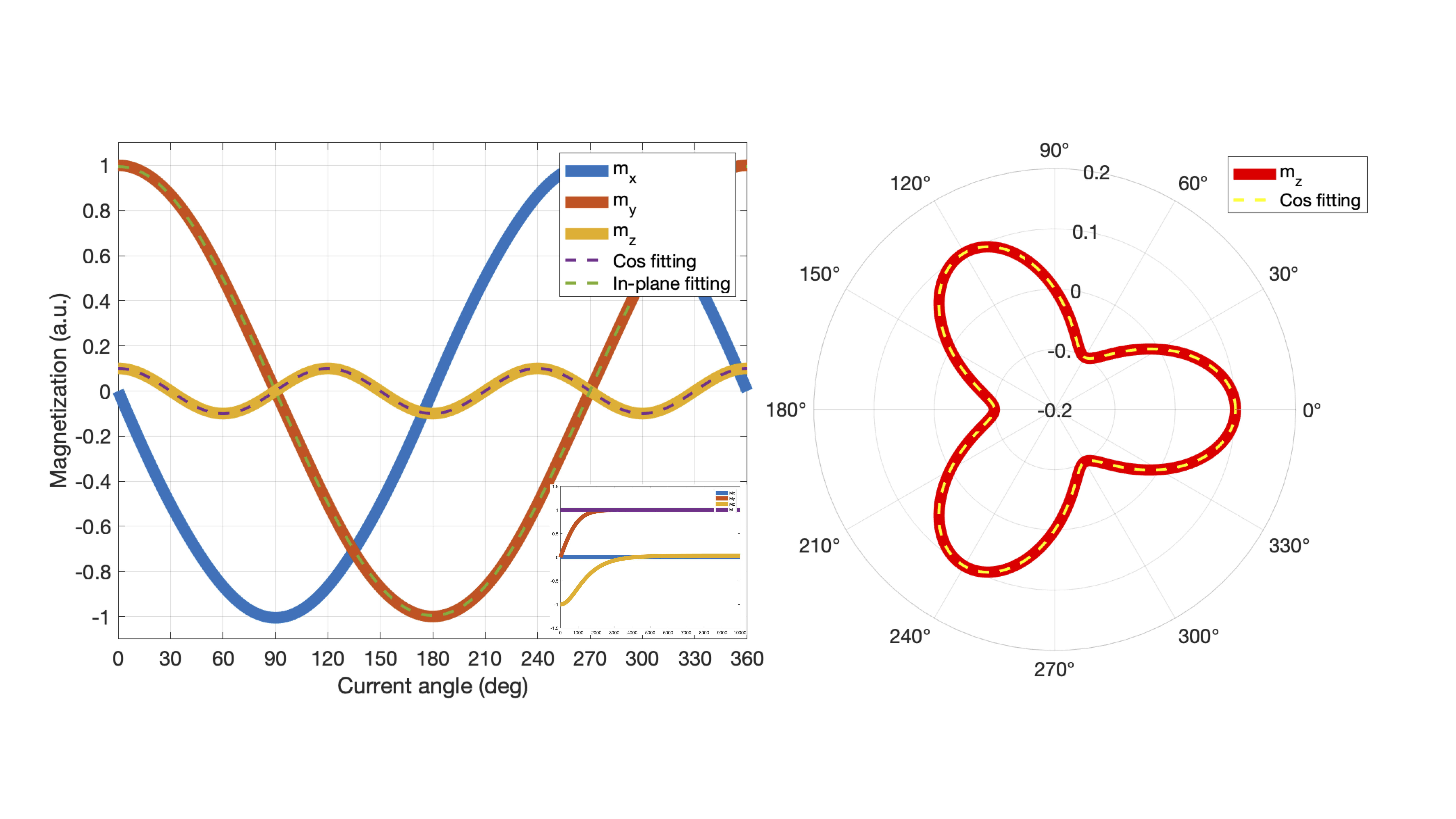


Fig S1. Numerical simulation of the magnetization under 3m torque. a) Current angular dependence of the magnetization at equilibrium with $\chi_{3m}/\chi_{DL}=-0.1$. Inset shows the evolution of magnetization at $\varphi=0$. b) Polar plot of $m_{z}$ with 3-fold symmetry.

The Cr_3_Te_4_ adopts a low-symmetry space group $P2/m$, while BST adopts $R\bar{3}m$. At the interface, both rotational symmetries will be lifted, and the net result is $Pm$. For point group $m$, after we consider the symmetry and the absence of $E_{z}$, we can write $\chi^{1}$ as:

$$\begin{aligned} \chi^{1}=\chi_{DL}X_{1}+X_{2} ,\#\left( AUTONUMLGL \backslash* Arabic \backslash e \right) \end{aligned}$$

where

$$\begin{aligned} X_{1}=\left( \begin{matrix} \hat{m}_{z} & 0 & 0 \\ 0 & \hat{m}_{z} & 0 \\ -\hat{m}_{x} & -\hat{m}_{y} & 0 \end{matrix} \right),\#\left( AUTONUMLGL \backslash* Arabic \backslash e \right) \end{aligned}$$

$$\begin{aligned} X_{2}=\left( \begin{matrix} {\alpha_{1}\hat{m}}_{y} & {\alpha_{2}\hat{m}}_{x} & 0 \\ {\alpha_{3}\hat{m}}_{x} & \alpha_{4}\hat{m}_{y} & 0 \\ 0 & \alpha_{5}\hat{m}_{z} & 0 \end{matrix} \right).\#\left( AUTONUMLGL \backslash* Arabic \backslash e \right) \end{aligned}$$

Unlike the case in point group 3m, $X_{2}$ now have more arbitrary coefficients $\alpha_{1}-\alpha_{5}$ because of lower symmetry. Following the same idea, we can calculate $m_{z}$ at the equilibrium:

$$\begin{aligned} m_{z}\left( \varphi\right)=m_{0}\frac{\cos\left( \varphi\right)\left( -\alpha_{1}\cos^{2} \left( \varphi\right) +\left( \alpha_{2}+\alpha_{3}-\alpha_{4} \right)\sin^{2} \left( \varphi\right) \right)}{\chi_{DL}}.\#\left( AUTONUMLGL \backslash* Arabic \backslash e \right) \end{aligned}$$

This final magnetization along z direction doesn’t have $2\pi/3$ periodicity in general. However, as mentioned in the main text, when three domains coexist, the system will restore the three-fold symmetry. This can be numerically calculated by adding up $m_{z}$ in all three directions:

$$\begin{aligned} m_{z}=&{(m}_{z}(\varphi)+m_{z}(\varphi+2\pi/3)+m_{z}(\varphi-2\pi/3))/3 \\ =&-\frac{{(\alpha}_{1}+\alpha_{2}+\alpha_{3}-\alpha_{4})}{4\chi_{DL}}m_{0}\cos\left( 3\varphi\right),\#\left( 14 \right) \end{aligned}$$

Now the combined $m_{z}$has the same periodicity as in the single 3m case.

We also solve the equation with numerical method. Here we choose several different values of $(\alpha_{1},\alpha_{2},\alpha_{3},\alpha_{4})$ for simulation. As shown in **Figure S2**, $m_{z}$ of a single domain generally does not have 3-fold symmetry. Actually, in many cases, it could deviate a lot from $\cos\left( 3\varphi\right)$(**Figure S2b** and **S2c**). However, the average $m_{z}$ of three domains that are 120° to each other will always have the 3-fold symmetry.


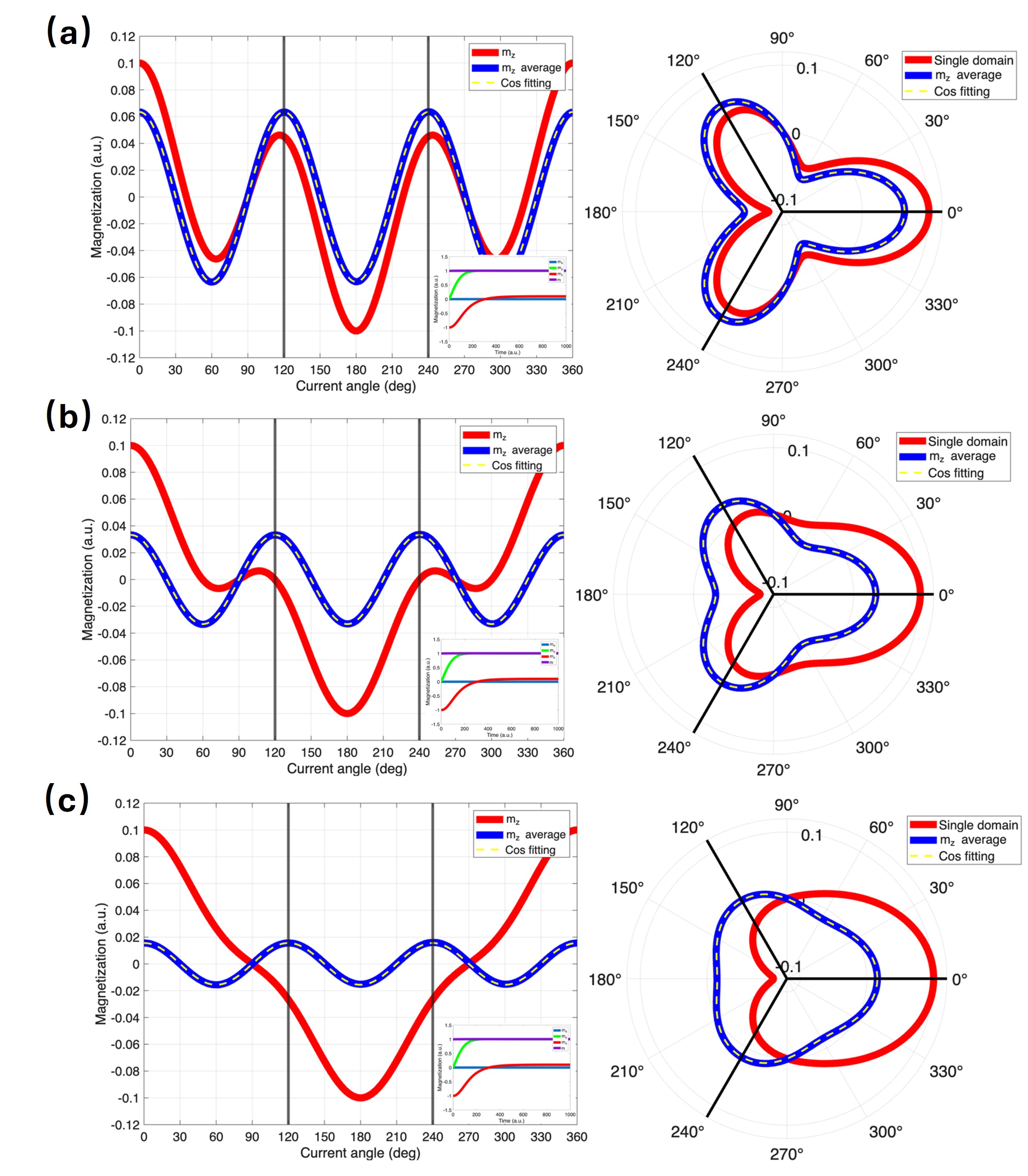


Fig S2. Numerical simulation of the z-magnetization under m torque with different values of $\left( \alpha_{1},\alpha_{2},\alpha_{3},\alpha_{4} \right).$ (a) $(-0.1, -0.039, -0.058, 0.057)$. Magnetization of all single domains already behaves similar to $\cos{3\theta}_{I}$, switching sign every 60°. (b) $(-0.1, -0.064, -0.0785, -0.109)$. When the current is aligned with one type of the domains, the other two (120° and 240°) have almost zero contribution. (c) $(-0.1, 0.033, -0.082, -0.088)$. When the current is aligned with one type of the domains, the other two have opposite magnetization, reducing the final combined z-magnetization.

**SI session 2: Topological Hall effect in Cr_3_Te_4_**

In the main text, we mentioned that there is a hump-like feature for the AHE measurement below 40 K. This phenomenon, also known as the topological Hall effect (THE), has been reported widely in the Cr_1+x_Te_2_ family [3,4,5]. In general, the hump-like feature can be simulated by summation of two distinct AHEs using formulas:

$$\begin{aligned} R_{AHE}=R_{s}\tanh\left( \frac{H-H_{c}}{2H_{c}}\cdot\ln\left( \frac{1+r}{1-r} \right) \right),\#\left( 15 \right) \end{aligned}$$

$$\begin{aligned} R_{H}=R_{AHE1}+R_{AHE2}+R_{OHE} ,\#\left( 16 \right) \end{aligned}$$

where $R_{s}$ is the Hall resistance after saturation, $H_{c}$ is the coercivity, r is a factor related to the shape, $R_{AHE1}$ and $R_{AHE2}$ are two distinct AHE and $R_{OHE}$ is the ordinary Hall effect that is proportional to the field. Larger r suggests a more square-like loop, while small r suggests an s-shape loop. As shown in **Figure S3**, the experimental data can be well fitted by the two-component model. Notably, the second component is more like an s-shape rather than a rectangular one, which means it is likely induced by in-plane anisotropy. Actually, it has been reported that the intercalated Cr_II_ atoms exhibit very small magnetic moments out of plane, indicating their moments almost entirely lie in-plane. Since the in-plane component would always be perpendicular to the applied current, this moment cannot break any out-of-plane symmetry thus has no contribution to the field-free SOT. The hump showing up only in actual field switching but not in SOT switching could be from this origin.


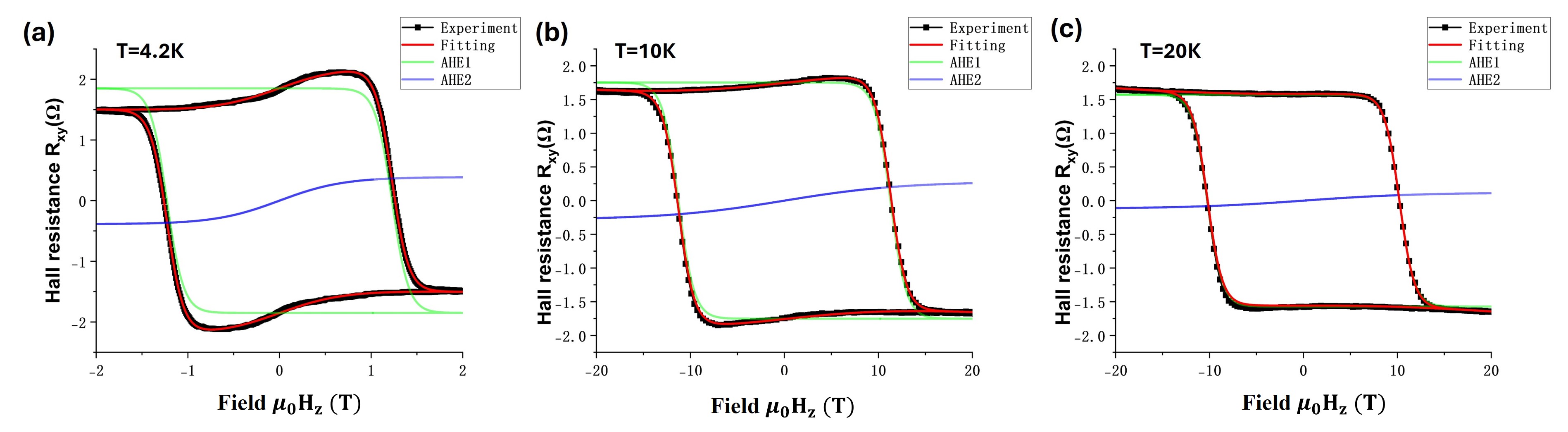


Figure S3. Fitting the experiment data with the two-AHE model at (a) T = 4.2 K, (b) T = 10 K, (c) T = 20 K. The first component AHE1 has a rectangular shape and contribute more to the total Hall effect, while the second component has an s-shape.

**SI session 3: Three domains of 2×1 sublattice**

During the STM measurement, within the typical scan areas of 10-20 nm, there are always grain boundaries showing up, suggesting high density of grains. For larger scans areas, such as 30-50 nm (but no longer in good atomic resolution), we can readily observe three or more domains coexist, giving an estimation of the typical domain size of 10s nm. Despite more than three domains coexist, the FFT clearly shows only three orientations.


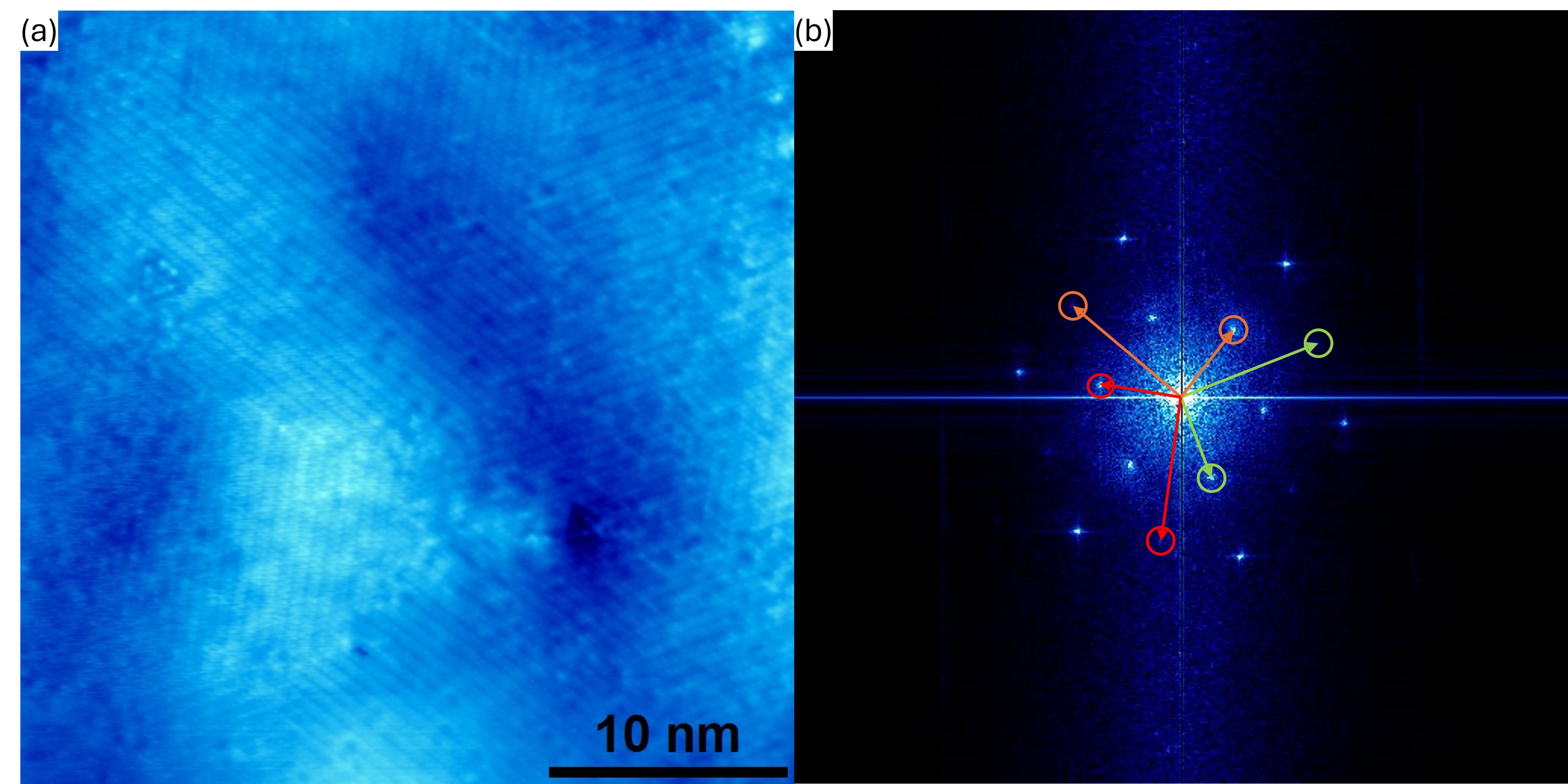


Figure S4. (a) STM image of a larger area where 2×1 cells with different orientations are randomly distributed. (b) FFT of the STM image. The arrows with different colors indicate three distinct 2×1 sublattices. They are exactly 120° to each other.

**SI session 4: SOT measurement under different bias field**

We have added the SOT measurement on the $\theta_{I}=0^{\circ}$ sample under different bias fields along the current direction at 4.2 K. As shown in **Figure S5**, the switching ratio reached 90% for -200 mT and 200 mT. This is expected since the external field breaks the symmetry and assists the switching. The hysteresis vanishes under 12.5 mT bias field and changes polarity across this field, an indication of the self-bias strength in our system.


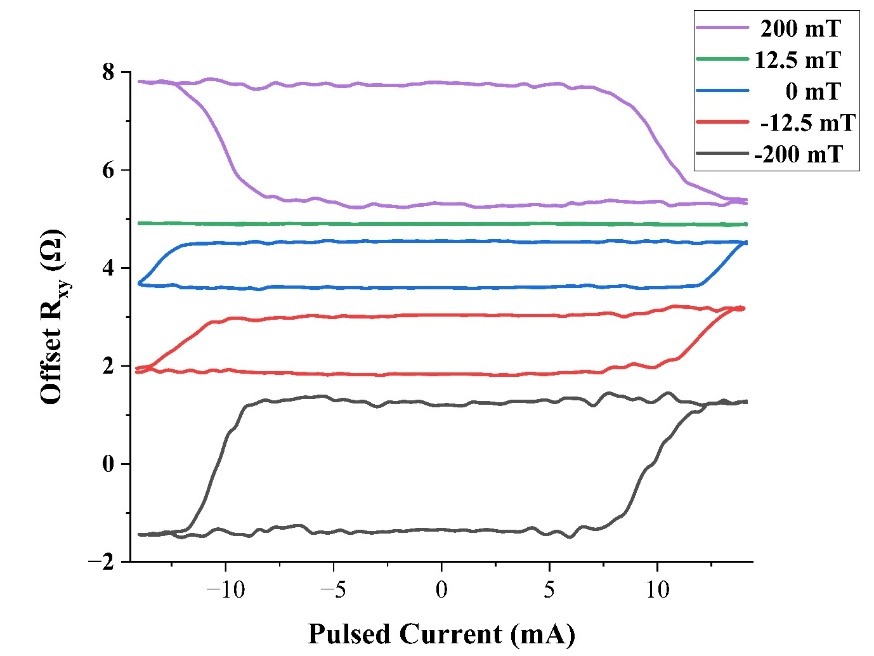


Figure S5. SOT measurement under different bias field along x direction (current direction.

**SI session 5: SOT in BST/CoFeB/MgO heterostructure**

In order to verify whether the surface state alone is able to achieve field-free SOT, we conducted SOT measurement on a BST (8 QL)/CoFeB (1 nm)/MgO (2 nm) sample under the same conditions ($T=4.2 K$, $\tau_{\mathrm{pulse}}=100 \mathrm{ns}$). While the CoFeB/MgO shows an out-of-plane anisotropy, the field-free SOT switching is missing (**Figure S6**). It switches normally under a bias field. To our best knowledge, there has not been any field-free SOT switching of PMA materials by BST family reported. Therefore, we conclude that despite the surface states already have 3m (C_3v_) symmetry and spin warping, the achievable perpendicular spin component is too weak to switch even soft materials like CoFeB. Our further reduced m (C_s_) symmetry allows for the strong deterministic PMA switching for hard magnets with H_c_ well over 1 T.


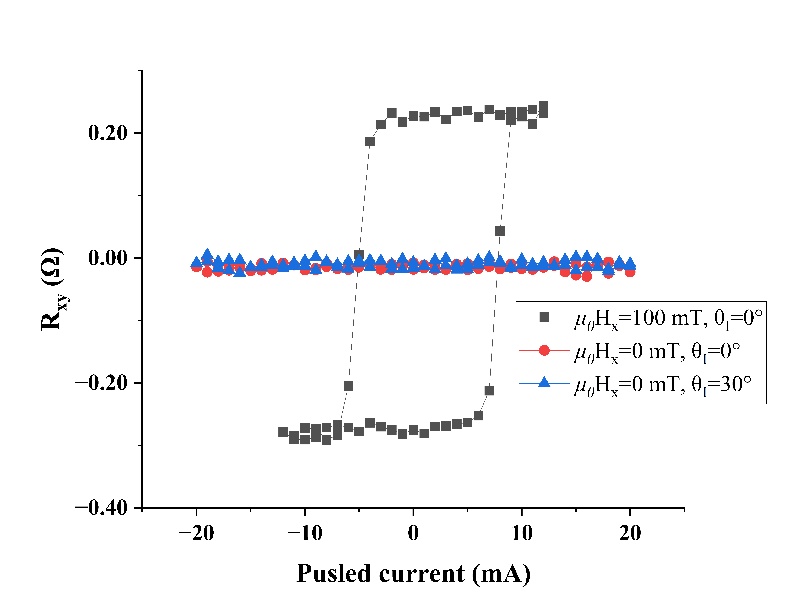


Figure S6. SOT measurement on a BST/CoFeB (1 nm)/MgO (2 nm) sample. The magnetization of CoFeB layer can be switched to the out-of-plane direction with a bias field of 100 mT along the current direction. However, there is no sign of switching with zero bias field for both the low and high-symmetry directions.

**SI session 6: SOT in Cr_3_Te_4_/Pt heterostructure**

We fabricated a Cr_3_Te_4_ (9 ML)/Pt (2 nm) sample and conducted the SOT measurement under the same conditions ($T=4.2 K$, $\tau_{\mathrm{pulse}}=100 \mathrm{ns}$). As shown in **Figure S7a**, no clear magnetic switching has been observed before device breakdown under 70 mA for both $\theta_{I}=0^{\circ}$ and $30^{\circ}$. We also did the field-assistant switching with a bias field $\mu_{0}H_{x}=$15 mT along the current direction (See **Figure S7b**). Within the breakdown limit, no switching was observed. The reported spin Hall angle of Pt is up to 0.08 [6] while the spin Hall angle tangent of BST in our case reaches 27.4. In fact, for most traditional Pt-based SOT devices in the literature, H_c_ of the magnetic layer to be switched is only up to several tens mT. The result could only prove that BST provides much larger damping-like torque than Pt which is critical to switch extremely hard magnetic materials (H_c_ > 1 T), before device breakdown reached in the given material system.

**
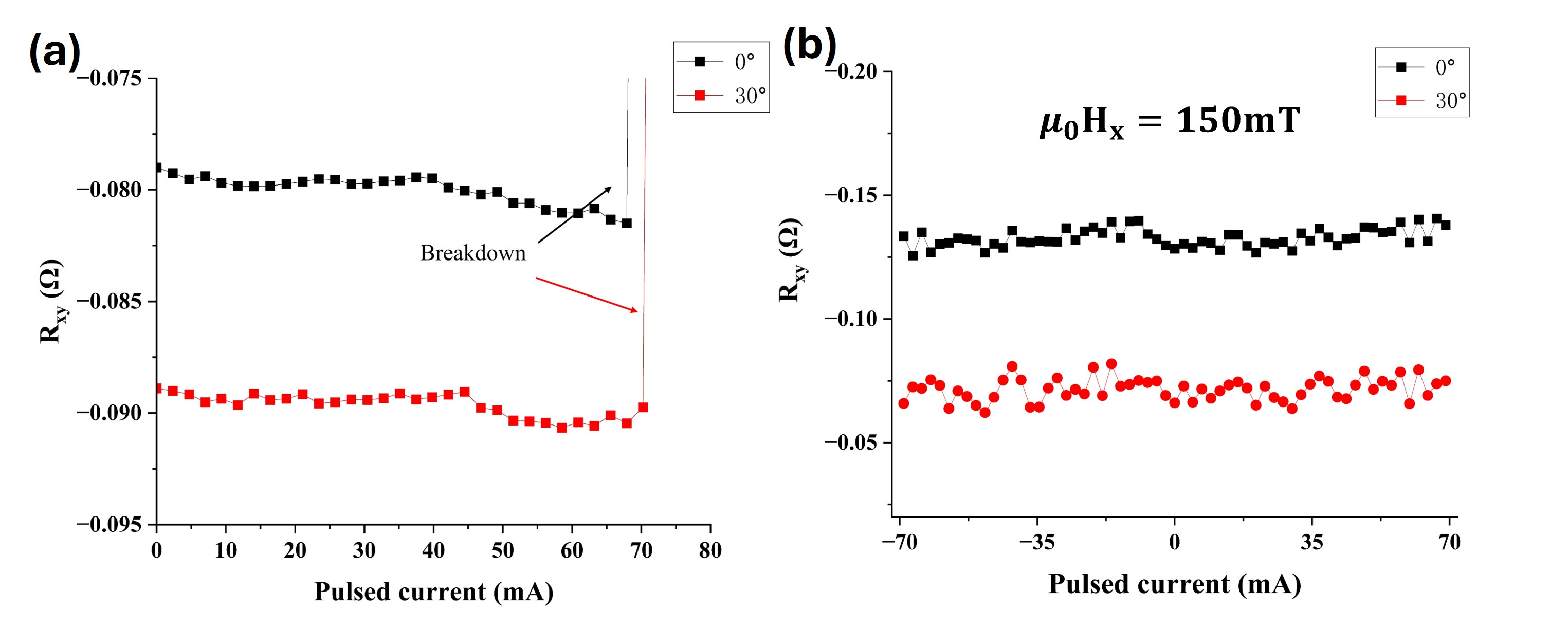
**

Figure S7. (a) Field-free and (b) 150 mT Field-assistant SOT measurement for Cr_3_Te_4_/Pt sample with pulsed current in different direction. Both samples show no clear hysteresis with or without bias field.

**SI session 7: Analysis of shunting effect**

**Figure S8** shows the resistivity vs temperature curves of 8 QL BST and 9 ML Cr_3_Te_4_ (CT) thin films and heterostructures by 4-point measurement. The resistivity of BST is higher than CT, but the thickness of BST in our device is larger than CT. The estimated shunt factor $\chi=\rho_{BST}t_{BST}/(\rho_{BST}t_{BST}+\rho_{CT}t_{CT})$ is around 0.68. Roughly 2/3 of the current flows through BST in the low temperatures.


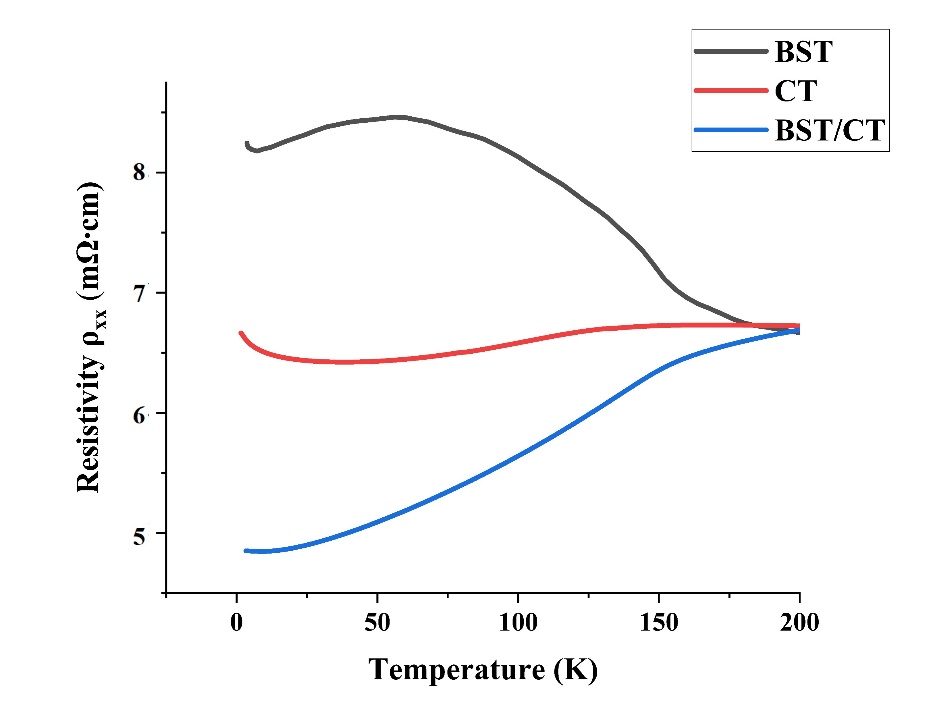


Figure S8. ρ_xx_-T curve of BST, Cr_3_Te_4_ and BST/ Cr_3_Te_4_.

**SI session 8: Characterization of BST**

**Figure S9a** shows the magnetoresistance vs applied magnetic field at 4.2 K. Where the percentage change of magnetoresistance (MR) is calculated by $MR\%=\frac{R\left( \mu_{0}H \right)-R\left( 0 \right)}{R\left( 0 \right)}\times100$. The magnetoresistance shows a pronounced weak anti-localization feature, which is a signature for two-dimensional topological insulator surface states, due to the inherently suppressed back-scattering [7*,*8]. This justifies the strong presence of the surface states. Next, we use SrTiO_3_ (STO) to gate the Fermi surface of the (Bi_0.5_Sb_0.5_)_2_Te_3_ thin film at the base temperature T = 4.2 K. The pronounced resistance maximum indicates the Fermi level crosses the Dirac point with gating (See **Figure S9b**). These are evidence that we have good control on the BST materials growth and properties.

**
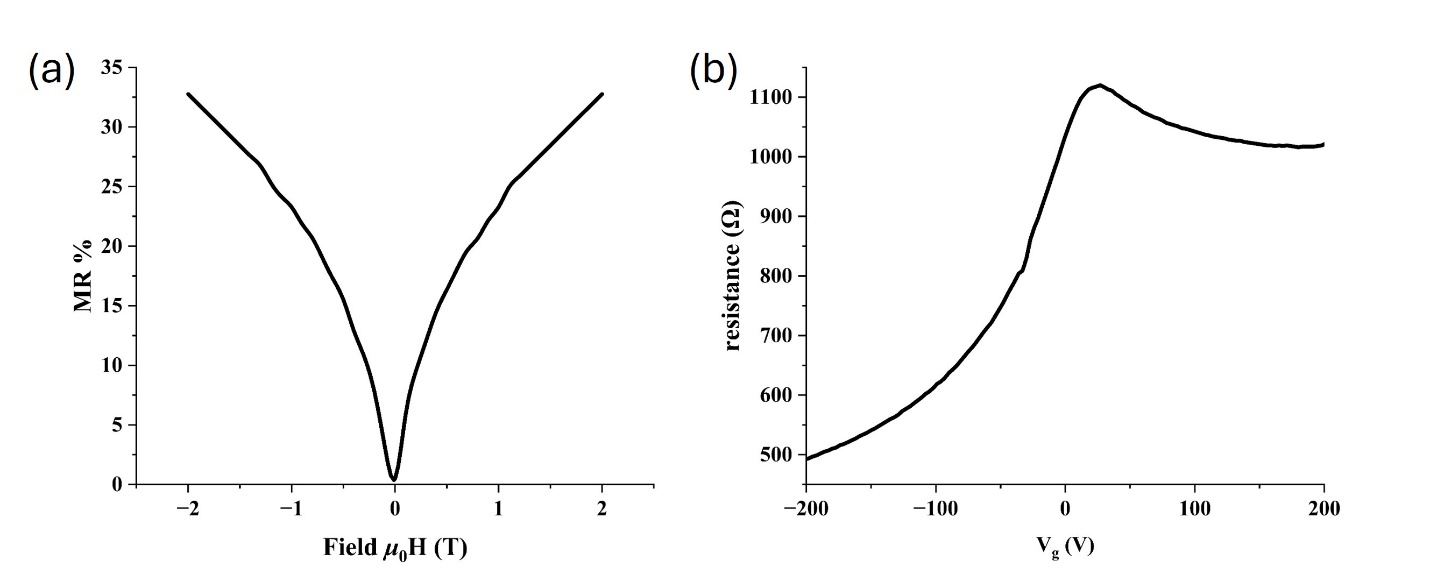
**

Figure S9. The characterization of the BST. (a) The normalized magnetoresistance curve. A sharp dip can be observed at zero field. (b) Resistance under different gating of BST by STO. The resistance maximum indicates the position Dirac point.

**SI session 9: Characterization of BST**

We also performed field-free SOT measurements on (Bi_0.25_Sb_0.75_)_2_Te_3_/Cr_3_Te_4_ and (Bi_0. 5_Sb_0.5_)_2_Te_3_/Cr_3_Te_4_ under the same conditions ($T=4.2 K$, $\tau_{\mathrm{pulse}}=100 \mathrm{ns}$). Compared to the (Bi_0.75_Sb_0.25_)_2_Te/Cr_3_Te_4_ case, the (Bi_0. 5_Sb_0.5_)_2_Te_3_/Cr_3_Te_4_ sample showed much lower switching ratio, while the (Bi_0.25_Sb_0.75_)_2_Te_3_/Cr_3_Te_4_ sample hardly switched.


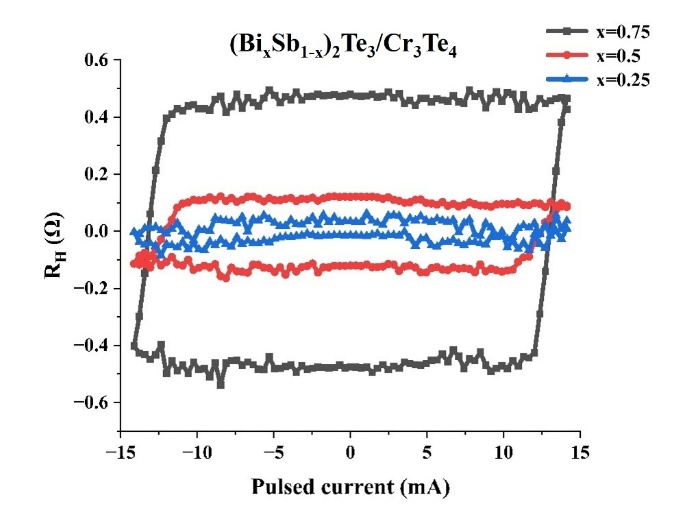


Figure S10. Field-free SOT measurement for different composition of (Bi_x_Sb_1-x_)2Te_3_ at $\theta_{I}=0$°. x=0.75 sample shows the clearest switching.

**Supplementary References:**

1. Železný, J., et al. "Spin-orbit torques in locally and globally noncentrosymmetric crystals: Antiferromagnets and ferromagnets." *Physical Review B* 95.1 (2017): 014403.
2. Li, Hang, et al. "Intraband and interband spin-orbit torques in noncentrosymmetric ferromagnets." *Physical Review B* 91.13 (2015): 134402.
3. He, Keke, et al. "Unconventional Anomalous Hall Effect Driven by Self-Intercalation in Covalent 2D Magnet Cr." (2024).
4. Burn, David M., et al. "Cr_2_Te_3_ thin films for integration in magnetic topological insulator heterostructures." *Scientific reports* 9.1 (2019): 10793.
5. Cho, Seong Won, et al. "Investigation of the mechanism of the anomalous Hall effects in Cr_2_Te_3_/(BiSb)_2_ (TeSe)_3_ heterostructure." *Nano convergence* 10.1 (2023): 2.
6. Wang, Tao, et al. "Large spin Hall angle in vanadium film." *Scientific reports* 7.1 (2017): 1306.
7. Bao, Lihong, et al. "Weak anti-localization and quantum oscillations of surface states in topological insulator Bi_2_Se_2_Te." *Scientific reports* 2.1 (2012): 726.
8. Gautam, Sudhanshu, et al. "Signature of weak-antilocalization in sputtered topological insulator Bi_2_Se_3_ thin films with varying thickness." *Scientific Reports* 12.1 (2022): 9770.
